# Evaluation of an Active Rehabilitation Program With Early Weightbearing and No Immobilization After Tibial Tubercle Distalization

**DOI:** 10.1177/23259671241287169

**Published:** 2024-11-11

**Authors:** Timo Rahnel, Frederick K. Weitz, Antti P. Launonen, Petri J. Sillanpää, Anna V. Hyvärinen

**Affiliations:** * Department of Orthopaedic Surgery, North Estonia Medical Centre, Tallinn, Estonia; † Pihlajalinna, Koskiklinikka Hospital, Tampere, Finland; ‡ Department of Orthopaedic Surgery, Tampere University Hospital, Tampere, Finland; § Department of Surgery, Tampere University Hospital, Tampere, Finland; ‖ Pediatric Surgery Department, Oulu University hospital, Oulu, Finland; Investigation performed at Tampere University Hospital, Tampere, Finland

**Keywords:** patella alta, tibial tubercle, osteotomy, patellar dislocation, active rehabilitation, complications, failure, stress fracture, MPFL

## Abstract

**Background:**

Abnormal patellar height (patella alta) has been reported to be one of the main predisposing factors for recurrent patellar dislocation, and it can be surgically corrected by distalizing tibial tubercle osteotomy (DTTO). Rehabilitation after DTTO often includes limitations on weightbearing and restrictions on knee range of motion by means of bracing, increasing the risk of slow progression of the rehabilitation.

**Hypothesis:**

An active rehabilitation program with no restrictions on weightbearing and range of movement would yield a low risk of postoperative complications and a fast recovery period.

**Study Design:**

Case series; Level of evidence, 4.

**Methods:**

Included were 102 consecutive knees in 80 patients who underwent DTTO between January 2010 and December 2017. In the majority of knees (89.2%), the patient underwent simultaneous medial patellofemoral ligament reconstruction. The mean age of the patients at the time of surgery was 19.39 ± 8.02 years, and 80.4% of the knees (82/102) were of female patients. The patients underwent an active rehabilitation program with immediate weightbearing as tolerated and active quadriceps and hip muscle exercises with no immobilization or bracing. The protocol was active (patient unsupervised), including daily exercises, as instructed by a physical therapist. Crutches were recommended for the first 3 to 4 weeks.

**Results:**

There were 3 acute failures of fixation (2.9% of knees) requiring revision surgery. In these cases, the patients had a fall, slip, or knee-twisting accident during the first 6 weeks after surgery. Two late failures characterized by tibial stress fracture at the distal part of the osteotomy level occurred at 2 and 3 months postoperatively and were considered unrelated to the early rehabilitation process. The stress fracture rate was 2%, and the overall DTTO failure rate was 6.9%. With the active rehabilitation program, adverse effects such as knee stiffness, arthrofibrosis, or delayed ability to perform daily activities were rare.

**Conclusion:**

An active rehabilitation program after DTTO was found to be safe and effective. Furthermore, the risk of failure related to surgical fixation strength and of later stress fracture was low.

Patellofemoral instability is a common condition in the young active population. Abnormal patellar height (patella alta) is reported to be one of the main predisposing factors for recurrent lateral patellar dislocation.^2,7,8,14,18^ Tibial tubercle osteotomy (TTO) is a commonly described surgical technique to correct patellofemoral disorders, especially patellar height abnormalities,^1,17^ and distalizing tibial tubercle osteotomy (DTTO) is a treatment option for the correction of excessive patellar height.^1,17^ Anteromedialization (ie, Fulkerson TTO) can be used for decompression of the patellofemoral joint with a symptomatic focal chondral lesion.^17^

As the medial patellofemoral ligament (MPFL) is the main passive patellar stabilizer against lateral displacement and is almost always injured during patellofemoral dislocation, simultaneous MPFL reconstruction is usually needed.^4,15,20^ In addition, other anatomic predisposing factors, such as high-grade trochlear dysplasia, may also require simultaneous correction.^16,17^

One of the aims of the postoperative rehabilitation protocol is to protect the fixation of the osteotomized tibial tubercle. Usually, rehabilitation after DTTO includes restricted weightbearing and restrictions on knee range of motion (ROM) using a brace 3 to 8 weeks after surgery. However, at present, there is a scarcity of data on rehabilitation programs after DTTO.

In the current literature, there is frequently a period of immobilization or restricted ROM as well as restricted weightbearing. On the other hand, previous studies have suggested that rehabilitation that is too accelerated causes an increase in complications. For example, Stetson et al^19^ reported a proximal tibial fracture rate of 2.6% during a 13-week period after Fulkerson osteotomy after changing the postoperative physical therapy protocol from partial to full weightbearing. As a result, they returned to a more conservative postoperative physical therapy regimen.

Cosgarea et al^6^ performed oblique (Fulkerson osteotomy) and flat osteotomies (Elmslie-Trillat or distalizing osteotomies such as those performed in the current study) on fresh-frozen cadaveric knees and found the mean load and total energy to failure was significantly higher in flat osteotomies. Thereafter, this finding was the rationale used to recommend postoperative bracing and restricted weightbearing until radiological proof of ossification at the osteotomy site in cases of oblique osteotomy. However, based on past literature regarding anterior cruciate ligament reconstruction and the discovered benefits of accelerated rehabilitation,^9,10,12^ the implementation of postoperative restrictions after DTTO may increase the risk of a slow progression to gaining normal movement of the knee and increase the subsequent risk for joint stiffness and patient discomfort.

In the present study, we described an active rehabilitation program for DTTO surgery and evaluated the recovery of ROM that can be achieved with this program as well as related DTTO failure rates, including acute loss of fixation strength or stress fracture and rates of other potential complications. We hypothesized that such an active rehabilitation program with no restrictions on weightbearing and range of movement could yield low rates of postoperative complication and a fast recovery period with acceptable rates of loss of fixation and stress fracture.

## Methods

**Figure 1.**
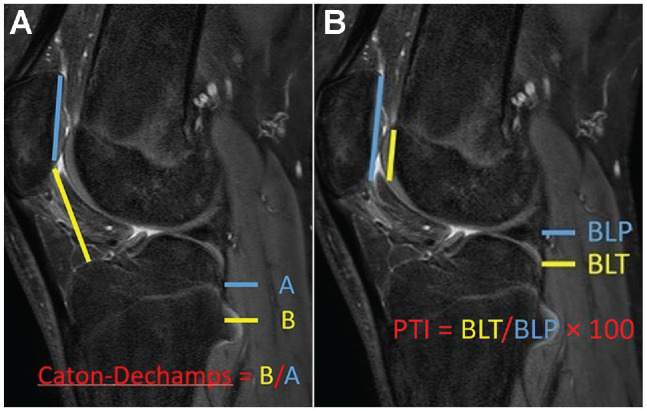
(A) The Caton-Deschamps Index, where *A* is the midline cartilage length of the patella and *B* is the length from the tibial plateau to the distal tip of the patellar cartilage. (B) The patellotrochlear index (PTI), where *BLP* is the midline cartilage length of the patella and *BLT* is the length of the trochlear cartilage that articulates with the patella.

Inclusion criteria for this study were knees that underwent DTTO for patellar instability or DTTO with simultaneous anteromedialization leading to decompression of a painful focal chondral lesion of the patellofemoral joint (n = 4) or anterolateralization to simultaneously correct overmedialization resulting from prior surgery years ago (n = 3). Patella alta was defined as a Caton-Deschamps index >1.2 or a patellotrochlear index <12.5% (Figure 1).^3,5^

**Figure 2.**
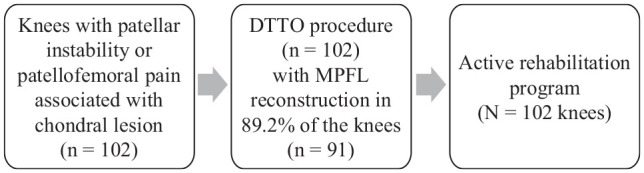
Flowchart of patient recruitment. DTTO, distalizing tibial tubercle osteotomy; MPFL, medial patellofemoral ligament.

Overall, 102 consecutive knees of 80 patients underwent DTTO by the senior surgeons (F.K.W. and P.J.S.) between January 2010 and December 2017. In the majority (89.2%) of the knees, the patients underwent simultaneous MPFL reconstruction, a soft tissue stabilizing procedure for recurrent patellar dislocation (Figure 2 and Table 1). Ethics committee approval for this study was waived, as studies in which the participants are not in contact/are not subject to intervention do not require the opinion of the ethics committee.

**Table 1:** Distribution of Accessory Procedures^*a*^

Procedure	No. of Knees
No additional procedure	6
MPFL reconstruction (+ arthroscopic debridement [n = 1] + arthroscopic meniscal fixation [n = 1])	50
MPFL reconstruction + femoral/tibial osteotomy	2
Trochleoplasty + MPFL reconstruction (+ quadricepsplasty [n = 1])	39
Patellofemoral arthroplasty (+ debridement, patellar component revision [n = 1])	3
Arthroscopic chondroplasty	2
Total	102

a MPFL, medial patellofemoral ligament.

DTTO was performed via a midline skin incision, using lateral to medial osteotomy with oscillating saw cut, and by removing the anterior tibial cortical bone block distal to the osteotomy, size-adjusted to allow sufficient distalization of the osteotomy to normalize the Caton-Deschamps and patellotrochlear indices. The fixation of the DTTO was performed using two 4.5-mm cortical screws (or three 3.5-mm cortical screws in the case of 1 knee).

Each patient underwent an active rehabilitation program with immediate weightbearing as tolerated by pain, without immobilization or bracing. Crutches were recommended for the first 3 to 4 weeks. The patients were allowed immediately after surgery to put full weight on the operated leg as tolerated. In practice, the patients did not tolerate full weightbearing in the first few weeks and therefore they were instructed to use crutches in the beginning and shift gradually to full weightbearing as tolerated after the first few weeks.

Postoperatively, the patients were seen by a physical therapist on the day of operation or on the first postoperative day; the physical therapist then instructed the quadriceps activation protocol for the following 2 weeks. The patients attended physical therapy sessions at 2, 4, and 6 weeks postoperatively, when ROM was also measured and recorded. Subsequent visits to the physical therapist were organized if needed, based on the individual needs of the patients. Most patients met their physical therapist at around 3 months postoperatively. The protocol for the first 2 weeks included 3 exercises; after 2 weeks, more exercises were added to the rehabilitation; then an additional 2 exercises were added at 4 weeks postoperatively; and 2 more were added at 6 weeks postoperatively (see the Supplemental Material, available separately, for the exercise protocol). Patients were instructed to perform the exercises independently at home on a daily basis. After radiographs and clinical follow-up at 6 weeks, active effort in knee extension was allowed while rising up or ascending stairs; light swimming and cycling exercises were also allowed.

The objective of the postoperative physical therapy protocol was to reach full extension with possibly a small loss of quadriceps strength and mass as well as to reach knee flexion angles that would allow cycling at 6 weeks postoperatively. Knee passive ROM was not restricted, and a tolerable amount of flexion was allowed, with patients typically achieving 90° of flexion at 3 or 4 weeks. The patients started with single-leg balance control exercises once tolerated, followed by more strenuous quadriceps strengthening, including stationary cycling and climbing stairs, at 6 weeks. Running on a flat surface was allowed at 3 months postoperatively or when radiographs indicated that DTTO had completely healed. Radiological and physical examination follow-up visits were organized at 6 and 12 weeks.

The primary outcome of the study was the recovery of normal knee ROM, and the secondary outcome was the complication rate. The preoperative and postoperative values were compared using the Student *t* test. Patient characteristics are presented with descriptive statistics, and binary data are presented as counts with percentages. Excel version 2408 (Microsoft) was used for analysis of the data.

## Results

The mean age of the patients at the time of surgery was 19.39 ± 8.02 years (median, 16.42 years; range, 10.08-55.5 years), and 82 of the 102 knees (80.4%) were of female patients. The mean Caton-Deschamps index was reduced from 1.19 ± 0.14 before surgery to 0.97 ± 0.14 after DTTO (*P* < .001). The mean length of distalization was 7.5 ± 2.6 mm. In some of the patients with patellofemoral instability, the patellar stability after the DTTO was assessed as good enough so that MPFL reconstruction, commonly combined with DTTO, could be avoided.

Follow-up visits to the surgeon or physical therapist included physical examination and assessment of knee ROM. Passive knee flexion was measured postoperatively. We studied recovery of the normal ROM after DTTO in patients with no previous surgery, those with loss of fixation postoperatively, or those diagnosed with hypermobility syndrome, as all of these factors potentially affect the recovery of ROM. The mean flexion values were 105° at 4 weeks, 120° at 6 weeks, and 135° at 12 weeks postoperatively, indicating a relatively fast recovery of normal flexion and no permanent stiffness. Flexion was considered normal when it was symmetrical compared with the contralateral knee. Data on extension measurements were available from 33 knees. In the vast majority of this subset of knees (29/33; 87.9%), full active and passive extension equal to the contralateral knee were achieved at 6 weeks postoperatively. In 3 knees, full active and passive extension were achieved later and documented in the subsequent follow-up visits. One patient had full passive but impaired active extension at 6 weeks, with no later records. Two patients developed arthrofibrosis.

**Figure 3.**
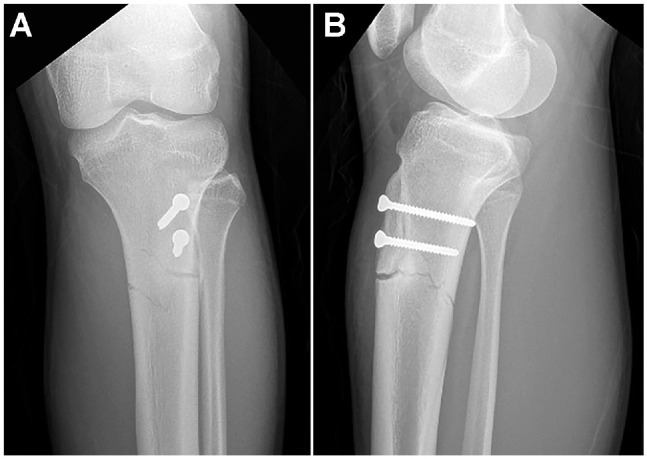
Tibial stress fracture. (A) Anteroposterior view; (B) lateral view.

Data on the 102 knees revealed 7 failures (6.9%) requiring revision surgery during the minimum 1-year follow-up period. These failures included 1 postoperative infection, 3 losses of fixation, 1 tibial stress fracture that required fixation, 1 conservatively treated tibial stress fracture, and 1 operatively treated failure of MPFL reconstruction. Five of these failures (6.9% of the total number of knees), including 3 losses of fixation (2.9%) and 2 stress fractures (2%; 1 patient treated nonoperatively) were considered directly related to DTTO. Early DTTO failure with a loss of fixation occurred in 3 patients who had a fall, slip, or knee-twisting accident during the first 6 weeks after surgery and was likely related to active rehabilitation, despite the fact that an injury was involved in all 3 failures. In the patients with loss of fixation, the distalization block split/fractured, including the tibial tubercle, slipped proximally near to or at its original position. Two late failures (2%) were tibial stress fractures at the distal part of the osteotomy level, which occurred at 2 or 3 months postoperatively; these were considered unrelated to the early rehabilitation process (Figure 3).

With the active rehabilitation program, the ability to walk without crutches occurred at a mean of 4 weeks after surgery, closely following the rehabilitation for isolated MPFL reconstruction. It is important to point out that none of the patients in the present study developed nonunion.

Those complications not directly related to osteotomy fixation are presented in Table 2. There were 2 failures of the MPFL graft (2%), 1 requiring revision surgery and 1 that was treated nonoperatively. One superficial infection (1%) was treated with antibiotics and 1 deep infection (1%) required revision surgery and a change of osteosynthesis material in addition to antibiotic treatment. Two other wound complications (2%) with no need for antibiotic treatment were detected. There were 2 arthrofibrosis cases (2%) that were related to previous multiple revision surgery and limited ROM before surgery (1 patient) or the inability of the patient to follow the rehabilitation program (1 patient who was then successfully rehabilitated). Importantly, in the latter case, the patient also underwent surgery for the contralateral knee due to severe patellar instability. In this case, the rehabilitation program and physical therapy guidance were further enhanced and arthrofibrosis was avoided. In 34 knees, there was a need for osteosynthesis material removal (33.3%). Patients were preoperatively informed of the potential need for the removal of material because a significant proportion of patients feel discomfort from the osteosynthesis materials. If discomfort was reported, patients were routinely offered the option of screw removal at the 3-month follow-up visit (Table 2).

**Table 2:** List of Complications^*a*^

Complication	Value
MPFL graft rupture	2 (2)
Superficial infection	1 (1)
Deep infection	1 (1)
Wound problem without antibiotic treatment	2 (2)
Removal of osteosynthesis material	34 (33.3)
Stress fracture	2 (2)
Arthrofibrosis	2 (2)
Failure of osteosynthesis due to trauma in the first 6 wk	3 (2.9)

a Data are reported as number of knees (% of total). MPFL, medial patellofemoral ligament.

## Discussion

The most important finding of the present study of 102 consecutive knees that underwent TTO followed by an active rehabilitation program, with immediate weightbearing but without immobilization or bracing, was that the patients regained normal knee ROM and the ability to walk without crutches after the first few weeks. The rate of stress fracture in this study was 2%, and the overall DTTO failure rate was 6.9%.

In our study, among 3 patients, loss of DTTO fixation strength caused by accidental injuries (eg, falling on stairs) occurred within 6 weeks of the surgery. Two tibial stress fractures at the distal part of the osteotomy level occurred at 2 or 3 months postoperatively; both were considered unrelated to the early rehabilitation process. The early failures were simple accidents and could have occurred despite the postoperative rehabilitation program. Our stress fracture rate of 2% is relatively low compared with previously reported data. For example, Luhmann et al^13^ reported a bony complication rate of 5.9% among a similar adolescent patient group (the mean age of the study cohort was 16.0 years [range, 12.2 to 20.2 years]). However, they used 3 different osteotomy methods. Of these, the method most similar to our DTTO technique had a stress fracture rate of 11.8%. Moreover, their postoperative rehabilitation protocol was clearly more conservative than the protocol in our study, with immobilization of the knees and restricted weightbearing for 6 weeks.^13^ Koëter et al^11^ reported a tibial stress fracture frequency of 3.3%, which is slightly higher than in our study despite postoperative immobilization with a cast for 6 weeks.

According to our experience, patients who have undergone previous multiple revision surgeries or who are incapable of independently following the rehabilitation program (eg, due to a neuropsychiatric condition) can be at increased risk for delayed recovery of knee ROM. Therefore, they should be informed of the risks and, if operated, may need an even more active, individually tailored rehabilitation program.

### Limitations and Strengths

The limitations of the present study include the retrospective analysis and the lack of a control group with a more traditional, slower rehabilitation regime. Furthermore, we did not have patient-reported outcome measures that would have allowed a more detailed analysis of the impairment of daily activities. The compliance with therapy was assessed during the control visits by the physical therapist and/or doctor. However, there was not a systematic way of reporting it in the patient records, which is a weakness of this study, related to its retrospective nature. As well, we were unable to find data from the patient records to present conclusive data on clinical outcomes and on the exact day the patients discontinued the crutches. Additionally, many patients first discontinued crutches indoors at home and somewhat later outdoors and/or during the school day, indicating a gradual discontinuation of crutch use.

A strength of the study is that all surgical procedures were performed by 2 senior investigators using the same technique. This provided consistency for the operative technique and the fixation method analyzed. No patients were lost to follow-up during the study period. The radiographic analysis was performed for all patients using a standardized method. Failure and complication analysis included a detailed assessment of patient history, including data on physical examinations, radiological studies, and revision surgery.

We are planning to carry out a prospective comparative randomized trial for confirmation of these results.

## Conclusion

An active rehabilitation program after DTTO can be considered safe and effective, with a low risk of immediate failure related to surgical fixation strength and low risk of stress fracture.

## Supplemental Material

sj-pdf-1-ojs-10.1177_23259671241287169 – Supplemental material for Evaluation of an Active Rehabilitation Program With Early Weightbearing and No Immobilization After Tibial Tubercle Distalization Supplemental material, sj-pdf-1-ojs-10.1177_23259671241287169 for Evaluation of an Active Rehabilitation Program With Early Weightbearing and No Immobilization After Tibial Tubercle Distalization by Timo Rahnel, Frederick K. Weitz, Antti P. Launonen, Petri J. Sillanpää and Anna V. Hyvärinen in Orthopaedic Journal of Sports Medicine
