# Supplementary material for: Evaluation of an Active Rehabilitation Program With Early Weightbearing and No Immobilization After Tibial Tubercle Distalization
Source: Orthop J Sports Med. 2024 Nov 11;12(11):23259671241287169. doi: 10.1177/23259671241287169 (PMC11555720; doi:10.1177/23259671241287169)
Supplement: sj-pdf-1-ojs-10.1177_23259671241287169 – Supplemental material for Evaluation of an Active Rehabilitation Program With Early Weightbearing and No Immobilization After Tibial Tubercle Distalization [file sj-pdf-1-ojs-10.1177_23259671241287169.pdf]

Personal Exercise Program

At 0-2 weeks postoperatively:

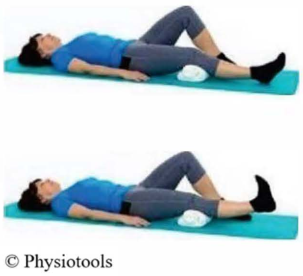

Knee End-range Extension in Supine

Lie on your back with one leg bent and the other leg straight.
Place a towel roll under the straight knee.

Bend your ankle and straighten the knee using your front thigh muscles.
Keep the back of your knee against the towel roll.

Keep the tension for a moment and then relax.

Repeat 10 times. Do the series three times.

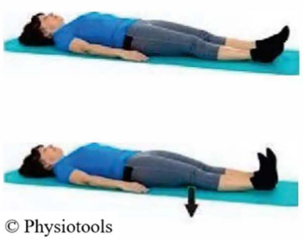

Isometric Knee Extension in Supine with Both Legs

Lie on your back with your knees straight.

Bend your ankles and press the back of your knees against the floor by by using your front thigh muscles. At the same time squeeze your buttock muscles.

Hold the tension for a moment and then relax.

Repeat 10 times. Do the series three times.

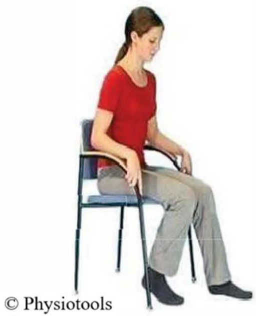

Seated Active Knee Flexion

Sit on a chair with your feet on the floor.

Bend your knee up to 90 degrees angle.

Repeat 10 times. Do the series three times.

At 2-4 weeks postoperatively:

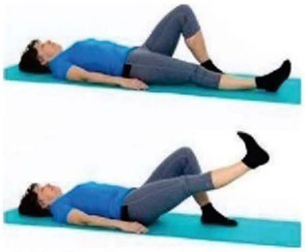

© Physiotools

Straight-Leg Raise in Supine

Lie on your back with one leg bent and foot on the floor, the other leg is straight.

Bend the ankle of the straight leg, contract the muscles of your front thigh, and lift the leg off the floor, keeping it straight.

In a controlled manner, return to the starting position.

Repeat 10 times. Do the series three times.

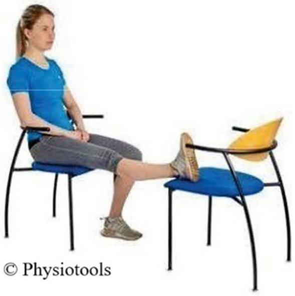

© Physiotools

Seated Knee Extension Stretch

Sitting on a chair, with the leg to be exercised supported on another chair as shown, let your leg straighten in this position.

Hold for 20 seconds. Repeat 10 times.

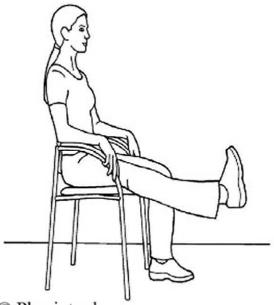

© Physiotools

Seated Active Knee Extension

Sitting on a chair, straighten then bend your leg.

Repeat 10 times. Do the series three times.

At 4-6 weeks postoperatively:

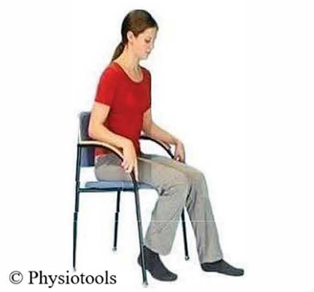

Seated Active Knee End-range Flexion

Sit on a chair with your feet on the floor.

Bend your knee as much as possible.

Repeat 10 times. Do the series three times.

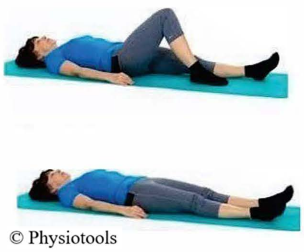

Active Knee Flexion in Supine

Lie on your back, with legs straight.

Bend your knee by sliding your heel towards your buttocks and return to the starting position.

Repeat 10 times. Do the series three times.

At 6 weeks postoperatively:

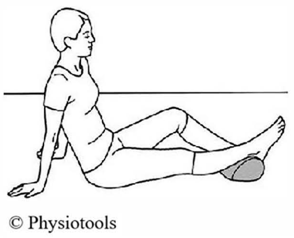

Place a towel roll under your ankle. Extend your knee as much as possible

Keep the tension for a moment and then relax.

Repeat 10 times. Do the series five times.

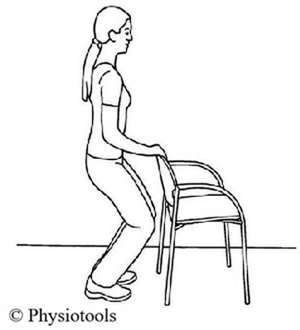

Take a grip with your hands on a chair.

Slightly bend your knees (about 30 degrees) and then extend your knees fully straight.

Repeat 10 times. Do the series three times.
